# Predictors for and Consequences of Acute Kidney Injury After Surgical Aortic Valve Replacement: An Observational Retrospective Study

**DOI:** 10.3390/jcm14207159

**Published:** 2025-10-11

**Authors:** Ivo Deblier, Karl Dossche, Anthony Vanermen, Wilhelm Mistiaen

**Affiliations:** 1 Department of Cardiovascular Surgery, ZAS Middelheim, Lindendreed 1, 2020 Antwerp, Belgium; ivo.deblier@zas.be (I.D.); karl.dossche@zas.be (K.D.); anthony.vanermen@zas.be (A.V.); 2 Faculty of Medicine and Health Sciences, Campus Drie Eiken, University of Antwerp, 2610 Antwerp, Belgium

**Keywords:** surgical aortic valve replacement, acute renal injury, predictors, survival

## Abstract

**Background/Objectives:** Acute renal injury (AKI) after surgical aortic valve replacement (SAVR) is a serious postoperative complication, associated with an increased need for resources and an increase in early mortality. **Methods:** In 2006–2017, 1548 patients underwent SAVR with or without an associated procedure. Preoperative and operative factors, as well as adverse postoperative events, were registered. The outcome was AKI defined by a decrease in the estimated glomerular filtration rate (eGFR) of at least 25%. Statistical analysis was performed with chi-square test and Student’s t-test. Significant factors were entered into a logistic regression analysis. AKI’s effect on long-term survival was determined via Kaplan–Meier analysis and Cox’s proportional hazard analysis. **Results:** AKI occurred in 447/1548 or 30.7% of the patients. Most preoperative cardiac and non-cardiac factors were associated with AKI. Prior endocarditis and a decreased eGFR were the dominant preoperative factors for early mortality, while a need for reintervention was the dominant postoperative event. AKI was also associated with prolonged surgical time and an increased need for resources. In patients who died within 30 days, AKI was not the sole complication. AKI also significantly reduced survival in the univariate analysis, revealing that AKI was a significant, independent predictor of survival, albeit the least strong. **Conclusions:** AKI is a serious postoperative complication associated with mostly non-modifiable factors. Postoperative AKI predicts reduced long-term survival.

## 1. Introduction

Acute kidney injury (AKI) after cardiac surgery is a serious postoperative complication. The rate of postoperative AKI varies depending on the definition of this event, patients’ comorbidity and age, and the type of surgery. The rate of AKI can be as low as 2% after elective cardiac surgery [1,2], and between 8.5% and 13.7% after isolated surgical aortic valve replacement (SAVR) [3,4]. This event can also be as high as 21.7% in some series of non-emergent cardiac surgery [5]. The need for postoperative dialysis occurred in 3.2% of patients suffering from AKI [5]. We previously observed a prevalence of AKI of 5.1% in a series of patients undergoing SAVR between 1987 and 2006. The predictors of AKI were chronic renal dysfunction, age above 80 years, atrial fibrillation, and a prior myocardial infarction [6]. An increase in age and comorbid conditions was observable before 2006 [7], and this increase continued in the following years [8]. In the current era, very elderly patients with symptomatic calcified aortic valve disease still undergo SAVR [9,10]. Hence, a further increase in the prevalence of AKI could be expected [7], with its undesirable consequences. Even a limited increase in the postoperative plasma creatinine concentration can increase the risk of postoperative mortality. This risk becomes very high if renal replacement therapy is needed [5,11]. Postoperative AKI is also associated with a reduced long-term survival [12]. Avoidance of AKI could improve the postoperative outcome if modifiable predictors can be identified and eliminated [11]. This study aimed to identify the predictors of AKI in a more contemporary series, the increased need for resources in patients suffering from AKI, and the long-term consequences of AKI on survival.

## 2. Materials and Methods

This study was a retrospective study of 1548 patients who underwent SAVR at a general hospital from 2006 to 2017. There was a follow-up of 12,018 patient-years. The preoperative and operative characteristics, as well as the postoperative outcomes, are listed in the tables. Patients who received a biological heart valve in the aortic position, with or without an associated procedure, were consecutively included. The exclusion criteria included having a mechanical valve or a valve in any other position, because their recipients have different patient characteristics and outcomes. Thirteen patients under chronic dialysis were also excluded. The inclusion and exclusion criteria, as well as the definitions of preoperative characteristics, the operative data, and the postoperative adverse events, were described in an earlier report. The preoperative factors under study were chronic kidney dysfunction (CKD), defined by an estimated glomerular filtration rate (GFR) of <60 mL/min; chronic obstructive pulmonary dysfunction (COPD), defined by a forced expiratory volume at 1 s of <80% of the predicted value; diabetes mellitus and hypertension, treated via diet and chronic medication; a history of histopathological documented malignancy; a history of a sudden neurologic ischemic event; an atrial fibrillation or conduction defect documented on ECG; the severity of the valve disease and left-ventricular function recorded using echocardiography; acute myocardial infarction recorded on ECG and by elevated heart enzymes; endocarditis, defined by the modified Duke criteria; coronary and peripheral artery disease (CAD, resp. PAD), documented as a >50% lesion on angiography; prior percutaneous or surgical procedures on the coronary arteries; permanent pacemaker (PPM) implantation; and the need for urgent SAVR (defined as a need for surgery during the admission at which the diagnosis was made) or emergent SAVR (defined as a need for SAVR within 24 h). The operative parameters included the aortic cross-clamp (ACC) time and cardiopulmonary bypass (CPB) time, expressed in minutes, the associations with CABG and mitral valve repair, and a procedure on the ascending aorta. The postoperative factors included the need for resources (the need for units of packed red blood cells, plasma derivatives, and thrombocyte concentrate, the duration of mechanical ventilation, the length of stay in the intensive care unit, and hospital stay) and adverse events, such as delirium with agitation, acute renal injury (a decrease in the GFR of at least 25%), clinical signs of atelectasis or pneumonia, confirmed by medical imaging, endocarditis, thromboembolism (sudden neurologic deficit or ischemia of a limb) of any severity, bleeding of any severity, new or recurrent atrial fibrillation, new or progress of a pre-existent conduction defect, low cardiac output (a need for mechanical support, prolonged intravenous inotropes, and low blood pressure with pulmonary or peripheral edema), and mortality. The outcome was an acute kidney injury, defined as a decrease in the eGFR within 24–48 h of at least 25% for grade 1, at least 50% for grade 2, and at least 75% for grade 3 [13,14]. The hospital mortality was defined as mortality within the hospital during the index stay or within 30 postoperative days. The long-term outcome was survival. These data were extracted from electronic medical records.

The statistical analyses included univariate chi-square test for categorical variables, Student’s t-test for continuous variables, and multivariate logistic regression analysis to identify significant predictors by entering those that were identified in the univariate analysis. Sensitivity analysis was performed by leaving out one potential predictor at a time while keeping the other in place to counter the risk for collinearity. Overfitting was avoided by not allowing more than one predictor per ten events. Reintervention was usually necessary to stop postoperative bleeding and could, therefore, be considered as an early event. Mechanical ventilation was routinely stopped soon after surgery. Because of these time relations, these events occur before AKI and can be considered as predictors. Kaplan–Meier analysis with log-rank test was used to assess the effect of AKI on survival. Cox’s regression model was performed as an alternative to propensity score match analysis and to assess the relative strength of its independent predictors. This study was approved by the ZNA Ethical Committee under the protocol N° 2656.

## 3. Results

### 3.1. Preoperative and Operative Variables with an Effect on AKI

A prevalence of 447/1548 or 30.7% for all grades of AKI was observed. Of these patients, 234 (52.4%) were in grade 1, 134 (30.1%) were in grade 2, and 76 (17.1%) were in grade 3. Renal replacement therapy (RRT) was required in 93 or 6.0% of the patients. The effect of preoperative patient characteristics was ranked according to the *p*-values, and with equal *p*-values, according to the chi-square test. These results are shown in Table 1. The dominant preoperative factor was CKD expressed as an eGFR below 60 mL/min. Age, diabetes, and other cardiovascular factors also had an effect. Patients suffering from AKI were 2 years older and had an estimated GFR of 9ml/min less. These patients had an 8% lower FEV1 and a 4% higher Euroscore II. The effect size or Cohen’s D for all variables ranged from small (0.200) to medium (0.500).

The operative factors in Table 2 were also ranked according to the *p*-values. Patients who suffered from postoperative AKI had significantly more associated procedures, and their incomplete revascularization rate was higher. The aortic cross-clamp (ACC) time increased by 6 min, and the cardiopulmonary bypass (CPB) time increased by 11 minutes., The absolute values for the effect size (Cohen’s D) are given alongside the *p*-values for the continuous variables. These effects were small to medium.

### 3.2. Postoperative Outcome and Need for Resources in Patients with AKI

Postoperative AKI was associated with all other abnormal lab exam results, increased need for resources (Table 3), and postoperative adverse events (Table 4). LOS (length of stay) in the ICU and postoperative stay increased by five and six days, respectively. The mechanical ventilation time increased by more than 24 h, and two extra units of packed red cells were needed. All these increases were significant (*p* < 0.001), with a medium (Cohen’s D between 0.500 and 0.800) or large effect size (at least 0.800).

All postoperative adverse events, except thromboembolism, were associated with AKI. Cardiopulmonary complications showed the highest degree of association. Although the mean transvalvular gradient across the bioprosthetic valve was significantly lower in patients with AKI, this difference was small, with a Cohen’s D value of less than 0.250. The 30-day mortality for patients with AKI was 75/445 or 16.9%. For patients without AKI, this value was 24/1101 or 2.2%, which was significantly lower (*p* < 0.001). The mortality rate significantly increased with the severity of AKI: 9.9% for grade 1, 21.6% for grade 2, and 27.6% for grade 3 (*p* < 0.001). Of the 93 patients who needed RRT, the mortality within 30 days was 46/93 or 49.5%. For patients without this need, this value was 53/1450 or 3.7% (*p* < 0.001). Of the patients who died within 30 days, only one patient had AKI as the sole postoperative complication.

A logistic regression analysis (Table 5) revealed that the dominant predictors for AKI were prior endocarditis and the need for reintervention (mostly because of bleeding), followed by preoperative chronic kidney disease (CKD). Active endocarditis was not a significant factor for AKI because of the low numbers. Chronic pulmonary dysfunction, diabetes, and other cardiac factors were also predictive. A sensitivity analysis, leaving out one of the predictors, revealed that the model was stable for each combination of the remaining eight predictors.

### 3.3. Effect of AKI on Long-Term Outcome

**Figure 1:**
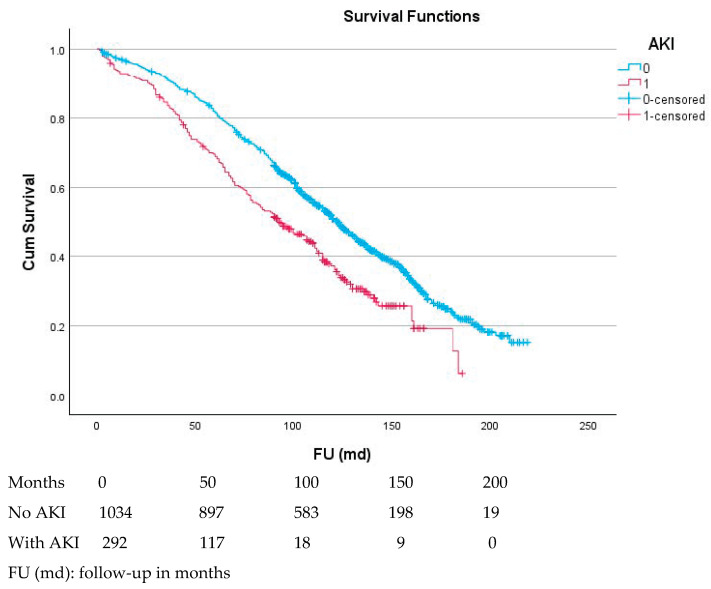
Relationship between the effect of acute kidney injury and survival.

The 5- and 10-year survival rates (Figure 1) of patients without AKI were 81.6 ± 1.2% and 51.3 ± 1.6%, respectively. These rates were significantly lower for patients with AKI, at 67.0 ± 2.5% and 35.2 ± 2.7% (*p* < 0.001). After a divergence in the first two years, the curves ran parallel. The effect of the AKI grade on survival was comparatively small. The 5-year survival rates of patients with a first-, second-, and third-grade AKI were 69.9 ± 3.3%, 66.3 ± 5.6, and 65.7 ± 8/8%, respectively. These values were not significantly different.

Cox’s proportional hazard analysis identified eleven independent predictors for long-term mortality (Table 6). Eight of these predictors were preoperative, one was operative, and two were postoperative. Age over 80 years was the most important predictor. Postoperative AKI showed a hazard ratio of 1.22 (1.01–1.47) for the latter, with *p* = 0.039.

## 4. Discussion

We observed that AKI occurred in over 30% of the patients undergoing SAVR. This was higher than the rate of 20–25% documented in a series involving three other cardiac centers [13] and much higher compared with our prior observation of 5% [6]. However, the current results were comparable to those found in another recent series [15] and could be explained by an increase in age and comorbid burden. This increase was already apparent before 2007 and continued to the end of the inclusion in 2017 [7,8]. The currently used diagnosis of AKI is based on changes in the GFR. We observed a significant effect of age, chronic renal and pulmonary dysfunction, and cardiovascular factors on postoperative AKI. Furthermore, almost all recorded adverse postoperative events were associated with AKI. An increased need for postoperative resources was also documented in patients suffering from AKI. The ICU stay was 5 days longer, while the postoperative hospital stay was 6 days longer. The need for blood products and PPM implantation was also significantly higher. The duration of mechanical ventilation was prolonged by 24 h. All observed laboratory values were significantly worse in patients with AKI. CKD was identified as the dominant factor in a univariate analysis, but preoperative endocarditis and a need for reintervention were the most relevant independent predictors of AKI in a multivariate logistic regression analysis. The observed mortality was almost eight times higher in patients with AKI. Except in one patient, AKI was never the sole cause of death in the first 30 days.

### 4.1. Risk Factors and Predictors for AKI

In a univariate analysis, a reduced preoperative GFR was the strongest factor in the development of postoperative AKI. In a prior published series, a preoperative GFR below 30 mL/min resulted in a 4.5 times increase in AKI [13] compared with patients with a higher GFR, and the estimated GFR was identified as the most significant factor [16]. A comparable effect was also seen after cardiac surgery in general [12]. CKD is more common in patients of 80 years and older [17], which makes age an important factor in the development of AKI. This was confirmed in the current and prior series [4,5,6,12,18]. One could assume that an age-related decrease in the GFR and renal blood flow within the glomerular capillaries plays a major role, but these effects vary widely between subjects. Associated structural changes include loss of renal mass, hyalinization of afferent arterioles, an increase in sclerotic glomeruli, and tubulointerstitial fibrosis [19]. Responses to vasoconstrictor stimuli such as the renin–angiotensin–aldosterone system (RAAS) are enhanced in elderly people. Sex seems to play a modulating role [19]. Male patients seem more vulnerable to AKI, since testosterone seems to activate the RAAS, while 17-beta-estradiol seems to lower it. The effect of male sex on the development of AKI was observed in some [11,13] but not all series [3,4]. Chronic pulmonary disease was also identified as a factor in the development of AKI in the current and past series [3,5,12]. A higher body mass index (BMI) has also been identified as a factor for postoperative AKI after cardiac surgery [3,4,15], but not in the current series, where a threshold of 30 kg/m^2^ was used. Obese individuals suffer from a higher degree of hypertension and diabetes. The latter conditions contribute to renal damage and glomerulopathy [15], which has been identified as a potent factor for AKI [3,4,5,12,13,18].

The need for concomitant CABG also increased the likelihood of postoperative AKI. This was also documented in earlier series and reviews, where a combined operation increased the risk compared with valve replacement alone or CABG alone [12,13,20]. However, prolonged ACC and CPB times, which are associated with concomitant procedures, were also implicated in the development of postoperative AKI [15,16,20]. A possible explanation is the increased rates of diabetes, hypertension, vascular disease, and CKD, which are well-known risk factors for coronary artery disease and, thus, for the need for CABG and longer CPB times [20]. Other cardiovascular factors that can increase the risk for postoperative SAVR include severe symptoms [13], prior cardiac surgery [13], prior congestive heart failure [18], preoperative myocardial infarction [6,18], preoperative atrial fibrillation [6,18], a need for non-elective surgery [11,12,13,18], and a low ejection fraction [5,13,18]. In current and past series, a need for prolonged ventilation [4,5], reintervention [5,18], and transfusion [3,5,11,12,18,21,22] was also associated with the development of AKI. A prolonged storage of red blood cells could cause structural cellular changes, which release pro-inflammatory molecules and lipids, promoting coagulation. Hypotensive states, which result from blood loss, should be avoided, since such states could promote the development of AKI [3]. The issue of a prolonged CPB time can be important for other reasons. The ratio between the actual lowest pump flow and the target pump flow could serve as a surrogate for low oxygen delivery, which could have an effect on the development of AKI [11]. Hypoperfusion of an oxygen-demanding medullary area of the kidney reduces oxygen-carrying delivery because of hemodilution, especially if the hematocrit level is below 25% [12]. A strategy of maintaining a hemoglobin concentration level at 7.5 g/dL seems non-inferior to the level of 9.5 g/dL during the CPB run and to the level of 8.5 g/dL in the postoperative period with respect to adverse outcomes, including AKI [23]. Since a prolonged need for mechanical ventilation and a need for reintervention because of bleeding could be considered early postoperative events, we reasonably expect these events to precede AKI. This allows us to enter these events into a multivariate analysis and identify them as predictors. Other factors include inflammation resulting from contact of blood with foreign material; manipulation and clamping of the aorta with consequent thromboembolism; ischemia–reperfusion damage; reduced cardiac output; hemolysis with release of free hemoglobin and free iron, promoting oxidative stress [3]; contrast nephropathy in the case of recent medical imaging; downregulation of vasodilatory mediators, such as nitric oxide; and upregulation of vasoconstrictive mediators, such as endothelin, catecholamines, and angiotensin II [3].

A comparison between SAVR and TAVI might be instructive with respect to AKI. A significant decrease in postprocedural AKI was observed over time, but its occurrence was associated with an increase in one-year major adverse events and mortality rates [24]. The occurrence of AKI had no major effect on transvalvular gradients after TAVT [25], which was also observed in the current series. AKI does not seem to be more common in patients of the ‘grey zone’ after TAVI compared with SAVR with a sutureless valve, according to a recently published meta-analysis [26]. The use of sodium–glucose cotransporter inhibitor-2 (SGLT2i) therapy seemed to protect against or at least mitigate AKI in diabetic patients with chronic kidney disease undergoing TAVI. Its effect could be mediated via improvement of the tubulo-glomerular feedback and the reduction in glomerular hyperfiltration via the vasoconstriction of the afferent arterioles. SGLT2i also decreased oxidative stress, the degree of inflammation, and overactivity of the sympathetic nervous system and the renin–angiotensin–aldosterone system [27]. AKI was associated with early mortality in the current and prior series [13,17]. A need for RRT also increased early death [3]. However, age, ACC duration, CPB, and age, which affected AKI in the current series, were also predictors for mortality [16]. In the current series, postoperative LOS in the intensive care unit significantly increased by 5 days, while the postoperative LOS increased by 6 days. Other series showed an increase in LOS of 2–4 days in grade-1 AKI, 4–10 days in grade 2, and 9–16 days in grade 3 [13]. In addition, patients suffering from AKI were significantly more likely to be readmitted to the ICU during their hospital stay [3]. In the current series, patients suffering from AKI had reduced long-term survival. After an initial divergence, both survival curves ran parallel. Eleven predictors for long-term mortality were identified using Cox’s proportional hazard analysis. Age was the strongest predictor for reduced survival, but seven other preoperative predictors were identified. A CPB time of over 120 min reflects a more complex operation and, hence, a more complex disease. The two postoperative predictors included delirium and AKI. The effect of AKI on survival was documented in an earlier series of patients undergoing cardiac surgery and was most evident within the first three months after surgery [2]. AKI of grade 2 or more affected survival to a large degree [14]. The effect of AKI on survival was confirmed in a meta-analysis in a non-cardiosurgical setting [28].

An important issue is a limited postoperative increase in plasma creatinine levels of between 0.06 and 0.30 mg%, which is below those corresponding to stage-1 AKI. In a nationwide observational series, this event was associated with an increased risk for 30-day all-cause mortality and a higher risk for long-term CKD and heart failure, but the incidence of these adverse events was lower compared with patients with a clinical AKI of grade 1 [29]. This so-called “subclinical AKI” could include some form of tubular kidney damage mediated by hemodynamic alterations and inflammatory responses [29]. The association between small creatinine increases and the development of heart failure may be attributed to a type-3 cardiorenal syndrome [29]. In patients suffering from a clinically overt AKI, these mechanisms would act more strongly. Readmission within 30 days because of heart failure after cardiac surgery was significantly higher in patients who suffered from overt postoperative AKI [30]. A meta-analysis showed that in patients from the general population who suffered an episode of AKI, the risk for long-term heart failure, acute coronary syndrome, and other unspecified major adverse cardiac events significantly increased. The rate of heart failure increased with the severity of AKI. Inflammation, activation of neuro-endocrine systems, mitochondrial dysfunction, metabolic acidosis, and high serum potassium levels could play a role in the development of these conditions. On the one hand, the renin–angiotensin system could contribute to renal vasoconstriction; on the other hand, this system could promote endothelial dysfunction, cardiac fibrosis, ventricular dysfunction, and heart failure [28].

### 4.2. Prevention of AKI

Maintaining adequate circulation during CPB and avoiding nephrotoxic agents are preventive measures against the development of AKI. Renal autoregulation is a mechanism to maintain renal blood flow and the GFR, even with oscillating pressures during the use of CPB. The interaction between maintenance of the systemic circulation and renal autoregulation determines the risk for AKI. A reasonable target for mean arterial pressure during CPB would be between 65 and 75 mm Hg, with a flow rate between 2.2 and 2.4 L/min/m^2^, which would ensure adequate oxygenation. The use of CPB should be kept as short as possible [12,31]. In patients with left-ventricular hypertrophy, which is often the case in aortic valve disease, an adequate preload can be maintained via judicious administration of intravenous fluids, but large volumes of isotonic saline should be avoided [12]. An intensive control of plasma glucose by keeping it between 80 and 110 mg% reduced the need for RRT [32]. However, strict control of plasma glucose in critically ill patients carries the risk of hypoglycemia [33]. Once AKI is established, an early initiation of RRT could prevent the development of metabolic acidosis, symptomatic uremia, hyperkalemia, and volume overload unresponsive to diuretics. The optimal timing to start RRT in severe AKI is uncertain, but a meta-analysis suggested that starting within 24 h resulted in a favorable outcome [34]. A routine use of prophylactic RRT after cardiac surgery in patients at risk for AKI could not be supported [12].

The limitations of this study are its retrospective nature. Selection bias was limited by the consecutive inclusion of patients undergoing SAVR. Postoperative GFR was estimated at its lowest level and not at a fixed time after surgery. However, almost all values of GFR were determined within the first 24 postoperative hours. The robustness of the model predicting AKI as an outcome was improved by sensitivity analysis. Cox’s proportional hazard analysis for long-term survival was used as an alternative to a propensity score-matching analysis. Access to digitalized medical files allowed a more detailed description of the patients compared with a nationwide series. Many patients resided in a nursing facility during the long-term follow-up because of the high mean age at inclusion, which limited access to the data needed with respect to adverse cardiac events.

## 5. Conclusions

AKI is a frequent complication after SAVR and carries a risk for early mortality. Its predictors were found to be prior endocarditis, the need for reintervention, chronic kidney disease, high age, chronic pulmonary dysfunction, diabetes, prior heart failure, prolonged mechanical ventilation, and the need for at least two cardiac surgical procedures. Most of these predictors are non-modifiable. The need for reintervention and for blood transfusion should be prevented as much as possible. Patients in need of such resources should be monitored more strictly. Even with the need for one or more additional procedures, the CPB time should be kept as low as possible. AKI carries a risk for decreased survival and could be considered a marker for a decreased organ reserve. Patients with a history of postoperative AKI should be monitored carefully for these reasons.

## Figures and Tables

**Table 1:** Effects of preoperative factors on AKI.

**Factor (Categorical)**	**AKI Absent (%)**	**AKI Present (%)**	***p***	**X^2^**
GRF < 60 mL/min	237/1016 (23.3)	205/369 (55.6)	<0.001	77.5
Congestive HF	250/1131 (21.1)	162/410 (39.5)	<0.001	49.0
Age > 80 years	307/1134 (27.1)	188/414 (45.6)	<0.001	39.2
NYHA III + IV	479/742 (64.6)	227/281 (80.8)	<0.001	35.1
FEV1 < 70% predict	159/1090 (14.6)	102/389 (16.7)	<0.001	27.4
Non-elective SAVR	178/1131 (15.7)	110/291 (26.7)	<0.001	26.7
Diabetes mellitus	249/1134 (22.0)	136/414 (33.0)	<0.001	23.7
Endocarditis	20/1134 (1.8)	28/414 (6.8)	<0.001	21.3
Pulmonary AHT	273/1084 (25.2)	139/385 (36.1)	<0.001	20.4
Atrial fibrillation	262/1130 (23.2)	150/412 (36.4)	<0.001	17.9
BMI > 30 kg/m^2^	279/1111 (25.1)	131/397 (33.0)	<0.001	16.0
Prior cardiac surgery	98/1134 (8.7)	64/412 (15.5)	<0.001	15.2
Hypertension	819/1129 (72.5)	327/410 (79.8)	0.002	
Coronary artery dis.	675/1134 (59.7)	281/414 (68.2)	0.003	
Acute myoc. infarction	169/1129 (15.0)	84/410 (20.5)	0.005	
Male sex	652/1134 (57.6)	274/414 (66.5)	0.020	
LVEF < 65%	357/687 (52.0)	154/258 (59.7)	0.046	
Digitalis	37/1127 (3.3)	22/410 (5.4)	0.060	
Ischemic neurol. event	154/1130 (13.6)	76/412 (18.4)	0.075	
Periph. artery disease	271/1131 (24.0)	117/389 (28.5)	0.096	
Prior PCI	155/1134 (13.7)	64/414 (15.5)	0.379	
Hyperlipidemia	681/1111 (61.3)	250/396 (63.1)	0.444	
Conduction defects	360/1129 (31.9)	150/412 (36.4)	0.788	
Malignancy	195/1128 (17.3)	68/409 (16.6)	0.968	
**Factor (Continuous)**	**Mean ± SD**	**Mean ± SD**	***p***	**Cohen’s D**
Euroscore	5.44 ± 6.12	9.47 ± 10.08	<0.001	0.538
GFR (ml/min)	68.7 ± 20.7	60.0 ± 23.2	<0.001	0.408
FEV1 (% predicted)	93.9 ± 21.5	85.7 ± 22.9	<0.001	0.377
Age (years)	75.0 ± 7.1	77.5 ± 6.4	<0.001	0.353
BMI (kg/m^2^)	26.5 ± 4.2	28.2 ± 4.7	0.026	0.204
LVEF (%)	61.4 ± 15.6	58.3 ± 16.1	0.007	0.193

AHT: arterial hypertension; AKI: acute kidney injury; BMI: body mass index; CABG: coronary artery bypass graft; CEA: carotid artery endarterectomy; FEV1: forced expiratory volume at 1 s; GFR: glomerular filtration rate; LVEF: left-ventricular ejection fraction; min: minutes; NYHA: New York Heart Association; PCI: percutaneous coronary intervention; Periph: peripheral; SAVR: surgical aortic valve replacement.

**Table 2:** Effects of operative factors on AKI.

**Factor (Categorical)**	**AKI Absent (%)**	**AKI Present (%)**	***p***	**X^2^**
CPB time > 120 min	437/1014 (43.1)	202/375 (53.9)	<0.001	15.8
Incomplete revasc.	98/1127 (8.7)	58/407 (14.3)	<0.001	12.8
Two or more procedures	651/1134 (57.6)	277/414 (67.2)	<0.001	11.6
ACC time > 60 min	556/937 (59.3)	220/316 (69.6)	0.001	
Concomitant CABG	640/1134 (56.6)	274/414 (66.5)	0.001	
Mitral valve repair	53/1134 (4.7)	32/414 (7.8)	0.005	
Concomitant CEA	22/1134 (1.9)	12/414 (2.9)	0.223	
Ascending aorta	100/1129 (8.9)	37/412 (0.0)	0.680	
**Factor (Continuous)**	**Mean ± SD**	**Mean ± SD**	***p***	**Cohen’s D**
ACC (min)	67.6 ± 23.0	74.6 ± 30.4	<0.001	0.411
CPB (min)	118.3 ± 42.1	129.3 ± 45.0	<0.001	0.266

ACC: aortic cross-clamp time; AHT: arterial hypertension; AKI: acute kidney injury; BMI: body mass index; CABG: coronary artery bypass graft; CEA: carotid artery endarterectomy; CPB: cardiopulmonary bypass time; FEV1: forced expiratory volume at 1 s; GFR: glomerular filtration rate; LVEF: left-ventricular ejection fraction; min: minutes; NYHA: New York Heart Association; PCI: percutaneous coronary intervention; Periph: peripheral; SAVR: surgical aortic valve replacement.

**Table 3:** Association of AKI with lab values and need for resources.

**Factor**	**AKI Absent (%)**	**AKI Present (%)**	***p***	**X^2^**
**Factor (Continuous)**	**Mean ± SD**	**Mean ± SD**	***p***	**Cohen’s D**
Reduction in GFR	3.0 ± 5.4	30.1 ± 15.6	<0.001	3.008
Lowest GFR	67.8 ± 21.0	31.2 ± 18.8	<0.001	2.143
Highest glucose (mg%)	165.4 ± 39.8	190.5 ± 59.2	<0.001	0.555
Nadir pO_2_ (mm Hg)	87.7 ± 22.4	76.2 ± 19.7	<0.001	0.528
Nadir hematocrit (%)	25.2 ± 3.4	23.8 ± 3.4	<0.001	0.428
**Need For Resources (Categorical)**				
Plasma derivatives	279/1111 (25.1)	151/395 (38.2)	<0.001	32.3
Thrombocyte conc.	101/1111 (9.1)	83/395 (21.0)	<0.001	28.7
Reintervention	28/1134 (2.5)	31/414 (7.55)	<0.001	21.9
PPM implantation	27/1134 (2.4)	24/414 (5.8)	<0.001	17.4
**Factor (Continuous)**	**Mean ± SD**	**Mean ± SD**	***p***	**Cohen’s D**
LOS-ICU (days)	1.8 ± 3.3	7.1 ± 12.2	<0.001	0.808
Postop. LOS (days)	8.7 ± 5.5	14.9 ± 12.5	<0.001	0.801
Mechan. vent. (hours)	9.2 ± 22.1	35.9 ± 93.5	<0.001	0.534
Units PCs	2.1 ± 2.7	4.0 ± 4.4	<0.001	0.608

LOS-ICU: length of stay in intensive care unit; PCs: packed cells; pO_2_: partial oxygen pressure; PPM: permanent pacemaker.

**Table 4:** Association of AKI with adverse postoperative events.

**Factor (Categorical)**	**AKI Absent (%)**	**AKI Present (%)**	***p***	**X^2^**
LCOS	49/1131 (4.3)	95/410 (23.2)	<0.001	121.8
Pulmonary complications	124/1134 (11.0)	146/414 (35.5)	<0.001	120.9
Mortality	28/1134 (2.5)	70/414 (17.1)	<0.001	113.9
Delirium	97/1128 (8.6)	91/406 (22.4)	<0.001	50.1
Bleeding	54/1134 (4.8)	70/414 (17.0)	<0.001	47.8
Conduction defect	195/1131 (17.2)	117/411 (28.5)	<0.001	19.7
Ventr. arrhythmias	32/1131 (2.8)	29/411 (7.1)	<0.001	10.8
Atrial fibrillation	423/1134 (37.4)	179/414 (43.6)	0.005	
Endocarditis	1/1131 (0.1)	3/411 (0.7)	0.041	
Thromboembolism	37/1134 (3.3)	20/414 (4.9)	0.142	
**Factor (Continuous)**	**Mean ± SD**	**Mean ± SD**	***p***	**Cohen’s D**
Mean TVG (mm Hg)	11.5 ± 5.0	10.3 ± 4.7	0.049	0.235
Peak TVG (mm Hg)	9.3 ± 8.9	19.3 ± 4.8	0.980	0.002

AKI: acute renal injury; conc: concentrate; glyc: glycemia; GFR: glomerular filtration rate; LCOS: low cardiac output syndrome; TVG: transvalvular gradient; Ventr: ventricular.

**Table 5:** Independent predictors for AKI identified by logistic regression analysis.

Predictor	OR	95% CI	*p*
Endocarditis	3.81	1.87–7.74	<0.001
Need for reintervention	2.97	1.62–5.44	<0.001
Chronic kidney disease	2.08	1.59–2.73	<0.001
Age > 80 years	1.91	1.46–2.50	<0.001
FEV1 < 70% predicted value	1.66	1.20–2.29	0.002
Diabetes mellitus	1.54	1.15–2.06	0.004
Congestive heart failure	1.67	1.25–2.22	<0.001
Mechanical ventilation > 8 h	1.81	1.38–2.37	<0.001
Two cardiac procedures	1.40	1.06–1.85	0.019

AKI: acute kidney injury; FEV1: forced expiratory volume at 1 s.

**Table 6:** Predictors of long-term mortality identified by Cox’s proportional hazard analysis.

Predictor	Hazard Ratio	95% CI	*p*
Age over 80 years	2.65	2.25–3.11	<0.001
Atrial fibrillation	1.55	1.30–1.84	<0.001
Chronic pulmonary disease	1.48	1.25–1.75	<0.001
Diabetes mellitus	1.39	1.11–1.58	<0.001
Prior malignancy	1.39	1.15–1.68	<0.001
Acute myocardial infarction	1.33	1.08–1.65	0.007
Atrioventricular block grades 1–2	1.41	1.07–1.85	0.015
Postoperative delirium	1.33	1.05–1.68	0.018
CPB time > 120 min	1.21	1.03–1.41	0.018
Peripheral artery disease	1.21	1.02–1.43	0.033
Acute kidney injury	1.22	1.01–1.47	0.039

CPB: cardiopulmonary bypass.

## Data Availability

These results were derived from a multipurpose database, from which several more publications will be derived. These data are not yet publicly available.

## Abbreviations

The following abbreviations were used in this manuscript:

ACC	Aortic cross-clamp
AHT	Arterial hypertension
AKI	Acute kidney injury
BMI	Body mass index
CABG	Coronary artery bypass graft
CEA	Carotis endarterectomy
CPB	Cardiopulmonary bypass
FEV1	Forced expiratory volume at 1 s
eGFR	(Estimated) glomerular filtration rate
ICU	Intensive care unit
LCOS	Low cardiac output syndrome
LOS	Length of stay
LVEF	Left-ventricular ejection fraction
NYHA	New York Heart Association
PCI	Percutaneous coronary intervention
PPM	Permanent pacemaker
RRT	Renal replacement therapy
SAVR	Surgical aortic valve replacement
SD	Standard deviation
SGLT2i	Sodium–glucose cotransporter inhibitor-2
TAVI	Transcatheter aortic valve implantation
